# Progress in Neurology

**Published:** 1901-03-23

**Authors:** 


### Progress in Neurology.

**Arsenical Poisoning in Beer Drinkers.**—It is to Dr. Reynolds of Manchester [1] that we owe the discovery of the cause of the epidemic of peripheral neuritis in the midland counties of England during the summer and autumn of 1900. The cases were thought at first to be due to alcohol, but symptoms such as erythema, keratosis, pigmentation, and herpes zoster were found associated with those which might have been due to alcohol. This led to the supposition that arsenic might be present in the beer —for practically all the patients were beer drinkers. The supposition was converted into a fact by the discovery of arsenic in the beer. These discoveries were immediately extended by Dixon Mann, Delépine, and Tattersall, who traced the contamination to certain sugars used in brewing, and thence to the sulphuric acid employed in making these sugars from cane sugar and starch. The original source was found to be the Spanish pyrites from which the sulphuric acid was made. The brewing sugars from one firm only were found to contain arsenic, but as this firm supplied no less than 200 breweries in the north of England and the midland counties, the epidemic was very widespread.

Quantitative analyses made by Delépine showed that the sulphuric acid contained 4 ozs. of arsenious oxide per gallon, the invert sugar ·25 part per 1000, the glucose ·8 part per 1000, and various beers from ·14 to ·3 grain per gallon.

The symptomatology is as follows: The patients have complained of one or more of the following symptoms: pains in the feet, hands and limbs, tingling in the fingers and toes, difficulty in walking, weakness in the hands and legs, rashes on the body, frontal headache, running of the eyes and nose, bronchitis and hoarseness, a tired-out feeling, shortness of breath, swelling of the feet, vomiting and diarrhœa. The aspect of the patient is often typical—the face is puffy, especially about the eyelids, the eyes are suffused and watery, and in some cases the conjunctives are œdematous and congested. The voice is often husky, and the walk is that of a patient with very sore feet, or somewhat unsteady, or else there is the high-stepping gait from paralysis of the dorsal flexors of the ankle. The skin lesions are very numerous and almost invariably present in one form or another. (*a*) Erythromelalgia was one of the commonest. The soles of the feet are crimson, as if stained with red ink, most commonly on the part of the sole which touches the ground in walking. The palms are similarly affected. Both soles and palms are tingling and burning hot and painful; and these signs are greatly intensified by heat. (*b*) Keratosis, which is a somewhat late manifestation, is frequently secondary to the painful erythema just described. This keratosis may be in a few isolated scaly masses or thickly covering the whole sole or palm, so that large scales are constantly being shed into the bed. (*c*) Erythema, either a scarlatiniform eruption, or more frequently a moribilliform rash running into scarlatiniform patches; in a few the change is so intense that bullæ form. (*d*) Pigmentation, which in most of the patients follows after many weeks the erythematous blush, which turns from red to copper-colour, and from that to bronze, and in severe cases almost to black. It is especially deep in tint round the neck, in the arm-pits, round the nipples, on the abdomen, round the genitals, and on parts where there has been pressure. There is no pigmentation of the mouth. In many cases after many weeks a browny desquamation of the pigmented skin takes place. (*e*) Herpes zoster. (*f*) In many cases the nails are affected, there is a transverse ridge distal to which the nail is thin and brittle.

Sensory affections have been present in practically all the cases. In the mildest they have merely consisted of paræsthesia and tinglings and burning and pricking sensations in the fingers and toes. In others there has been an associated numbness, though not a total loss of sensation. In a large number of cases (but only if there was some loss of power) there was tenderness on pressure of the muscular masses of the arms and legs, either slightly marked, in others very intense.

Motor symptoms were present in greater or less degree in 70 per cent. of the cases. In the slighter cases there was only slight loss of grip and slight affection of the gait, whereas in more marked cases there was a total paralysis of the affected muscles with marked atrophy. The small muscles of the hands, the muscles of the forearm, especially the extensors, and in severe cases all the muscles of the arm were affected. In the legs the interossei and the anterior tibial and peroneal muscles became paralysed and wasted, so that the toes were flexed and the foot dropped at the ankle into a position of equino-varus. The calf muscles were afterwards affected, at the same time as those of the thigh, accompanied with loss of knee jerk. The trunk muscles became weak, and the patients could not raise themselves in bed, and in advanced cases the diaphragm became paralysed. There was no paralysis of the sphincters, except in the most advanced cases, in which the incontinence was mostly due to the mental weakness. The intercostal and cranial muscles were unaffected. Many of the patients were inco-ordinate in their movements, but there was never any real resemblance to the ataxy of tabes. In many of the cases of advanced paralysis a peculiar mental condition was found—a total loss of memory of time and then of place, accompanied by a loss of initiation of ideas.

In the majority of cases there was heart failure, in the most marked ones with dilatation of the left side of the heart. The principal cause of death was cardiac failure In 25 per cent. of cases œdema was present, affecting the feet only or nearly the whole body. There was a fair amount of ascites, but no great amount of pericardial or pleural effusion. Thus arsenic will seriously affect the heart muscle. The respiratory mucous membrane, like the skin, was irritated by the arsenic. In the early stage there might be running from the eyes and nose, congestion of the fauces, marked congestion and thickening of the vocal cords and very pronounced bronchitis. Not a few showed signs of phthisis, with fairly rapid breaking down of the lung tissue.

In many of the cases digestive troubles were the first signs. The tongue in an early stage had a typical thin, white, silvery coat, as if it had been brushed with lunar caustic; in later severe cases it was brown. Vomiting, quite sudden and copious, was a marked feature, sometimes occurring after meals, or immediately after a pint of beer had been taken. Some patients complained of diarrhœa. Cirrhosis of the liver has apparently been set up in a few cases. In many cases there was a trace of albumen in the urine, but quite possibly this was secondary to heart failure. In patients who had been drinking quite recently arsenic was discovered in the urine by Dixon Mann. In the early stages of several cases the temperature was raised for a week or more, the other signs of arsenical poisoning being fairly acute. From the above account it is clear that arsenic is almost certainly a cumulative poison, and a poison which affects the skin, respiratory and intestinal mucous membranes, the nerve trunks both motor and sensory, the voluntary and cardiac muscle and the liver. The sequence is, first, digestive symptoms; second, laryngeal catarrh, bronchitis and acute skin affections; third, disturbances of sensibility; fourth, motor paralysis, pigmentation and keratosis.

The course of the disease is slow; the gastric, coryzal and laryngo-bronchial symptoms first pass away, then the acute skin lesions, whilst the chronic skin lesions and the nerve paralyses last for very many months. The mode of death in most cases is from cardiac failure. The diagnosis, once the possibility of arsenic poisoning is recognised, is easy. In the early stages it is possible to mistake the disease for measles or scarlet fever, and in the latter for Addison's disease. Once the intake of poison is stopped, the treatment is a matter of dealing with symptoms; in doing so great attention must be paid to the cardiac weakness. For the pains morphia may be used, and the burning pains in the hands and feet may be relieved by spirit lotion. The treatment of the neuritis does not differ from that which is already well known.

The discovery by Reynolds of contamination of beer by arsenic led to the publication of many cases of arsenic poisoning from beer drinking in the Midlands. Buchanan,[2] for instance, has described cases occurring at Liverpool; Bowser[3] similar ones at Penrith, but hitherto all the cases described have been traced to the one firm in Liverpool who used arsenic-containing sulphuric acid in making invert sugar and glucose.

Sturrock[4] has called attention to the number of beer-drinkers in whom the poison seems to have missed the peripheral nerves, and only attacked the heart or liver. In the hepatic cases, the complaint of the patient has been swelling of the abdomen, pain in the liver region, in some cases so acute as to simulate biliary colic, and in half the cases, blood in the stools. On examination one has found an enlarged, hard, tender liver extending about two inches below the costal margin, considerable ascites without œdema, and in some cases icterus. With rest in bed and simple treatment the ascites has disappeared with great rapidity, and within a fortnight the liver has resumed its former limits. The condition might be ascribed to beer-drinking, but the incidence of the considerable number of cases at this time, and the fact that the quantity of beer drunk seems in no case to have been excessive, make one imagine that the cause must be identical with that which produced such a large crop of cases of peripheral neuritis.

Bury[5] has shortly discussed the diagnosis of arsenical from alcoholic peripheral neuritis—a matter of great interest, seeing that in many respects the disorders produced by these two poisons are identical. One characteristic difference is that alcohol does not produce any cutaneous lesions. Again, in alcoholic cases there is no evidence of irritation of the respiratory and digestive tracts, in anything like the degree as is found in arsenical cases. In regard to the neuritis, hyperæsthesia of the skin and muscles, though common in alcoholic neuritis, is more constant and more severe in the arsenical cases. Erythromelalgia is only very rarely present in alcoholic cases. Ataxia, again, is very common in the arsenical cases. (This is in contradiction to Reynolds' opinion.) In the acute stage a water-bed is of great value to a patient. For the relief of tender nerves and muscles there is nothing better than warm fomentations. In the acute stage strychnine must not be given or massage employed. After subsidence of the acute stage, massage, electricity, and the hypodermic administration of strychnine are of the greatest value.

Some of these cases of arsenical poisoning at Chester were at first attributed to beri-beri by Newall and

Prytherch. The opposite suggestion was subsequently made by Ronald Ross,[6] that many cases have been attributed to beri-beri in the East, when the true cause was perhaps arsenic. Natives of India and Burma are very fond of lemonade (manufactured from European chemicals), tinned and bottled fruits, into which arsenic may easily get. The sudden improvement occurring in most cases of beri-beri, after removal from the house where they contracted the malady, is certainly more suggestive of intoxication than of infection.

Tunnicliffe and Rosenheim[7] have made the suggestion that arsenic has not been the only poison at work in the Manchester epidemic. They were struck with the apparent incongruity between the doses of arsenic taken and the effects produced. Now selenium occurs together with arsenic in nearly all pyrites and sulphuric acid prepared from them. And the authors found on investigation that commercial oil of vitrol (made by the firm who had sent out the arsenic-containing sulphuric acid) contained 0·2 per cent. of selenium. Now selenium compounds are highly poisonous, and their physiological behaviour is practically identical with that of arsenic. Hence it is probable selenium compounds have played a definite though subsidiary rôle in the recent arsenic epidemic.

[1] Lancet, Jan. 19th, 1901. [2] Ditto. [3] Brit. Med. Jour., Dec. 22nd, 1900. [4] Ditto. [5] Brit. Med. Jour., Dec. 8th, 1900. [6] Ditto. [7] Brit. Med. Jour. Feb. 2nd, 1901.

